# Collaboration with the mesh industry: who needs who?

**DOI:** 10.1007/s00192-016-3075-8

**Published:** 2016-07-13

**Authors:** Jan-Paul Roovers

**Affiliations:** Department of Gynaecology, Academic Medical Center, University of Amsterdam, Meibergdreef 9, 1105 AZ Amsterdam, The Netherlands

**Keywords:** Urogynaecology, Industry, Collaboration, Mesh

## Abstract

Professional physician organisations should be the coordinators of all stakeholders involved in the field of urogynaecology. That means that, together with representatives of the patients (“patient organisations”) they develop a priority agenda. This agenda involves the topics that need the highest level of attention, either because knowledge in this area is insufficient, or because existing knowledge is not optimally implemented. The next step is to discuss this list with representatives of industry, patient representatives, insurance companies and organisations with an intention to donate money to women’s health. Together, we can design a roadmap for the coming years that involves the top priority topics that need to be dealt with within the available budget. The organisations facilitate the interaction between the different stakeholders, communicate the timetable of these actions not only to their members, but also to society at large.

## Introduction

Just a few weeks ago, Endo International decided to stop selling the portfolio of Astora women’s health products and close down the business (personal communication). Astora, formerly known as the AMS women’s health division, was the new corporate name for the business following the sale of the AMS men’s health division and the AMS brand name to Boston Scientific in mid-2015. Physicians, patients, professional organisations, competitors and lawyers were all surprised by the sudden decision undertaken by Endo.

## How did it all start?

In the 1990s, physicians started to use mesh in vaginal surgery to improve outcomes. Interest from industry followed and in the early 2000s, the first commercial vaginal mesh kits were introduced onto the market. The initial (underpowered) studies with a short follow-up still showed superior anatomical outcomes [1]. The results were sufficient to generate much attention among gynaecologists and urologists, who were not only motivated to improve outcomes in their patients, but were also interested in adopting new surgical procedures in their existing, mostly narrow, surgical portfolio.

Professional organisations gained significant financial benefits from the introduction of vaginal mesh surgery. Sponsorship fees and financial support from industry for congresses increased rapidly. Congress registration numbers in turn increased rapidly because of industry sponsoring doctors’ attendance at international meetings. In addition, professional organisation sessions and meetings sometimes lacked their traditional scientific rigor and allowed podium presentations of poorly designed studies including some with very preliminary results.

## What happened after that?

Like all innovations, drawbacks become clear at the moment at which implementation in a wider sphere occurred. Physicians with less experience in pelvic floor surgery started to perform vaginal mesh surgery on less appropriate patients (i.e., patients at a higher risk of complications or poor outcomes). We know that these less experienced physicians never blame themselves when a procedure has poor outcomes, but instead blame the surgical technique. In my opinion, it is easy to consider the physicians to be responsible for the overly rapid introduction of new surgical procedures. We know that there has been almost no improvement in surgical outcomes of native tissue repair procedures over many decades. Given the high number of procedures performed for recurrence, it is not surprising that pelvic floor surgeons quickly placed vaginal mesh surgery somewhere in their treatment protocol. For professional organisations, these rapid and large-scale changes are impossible to control. Some were very responsive in designing public statements in reaction to the FDA notifications and public opinion.

What the professional organisations could have done differently is to explain that vaginal mesh surgery may have had an overly aggressive start, but that there was a second chance to make a better start. Unfortunately, their response was the opposite. Again, they did not follow science, and podiums were provided to those who were most critical of mesh, were never involved in studies evaluating mesh, and admitted that they took more mesh out than they ever had placed. The same happened with scientific journals, in which it was easier to publish papers that were critical of mesh than papers supporting the use of mesh.

In my opinion, the use of mesh in vaginal prolapse surgery is better supported by data than sacrospinous fixation, laparoscopic sacrocolpopexy or the McCall procedure, all far more frequently performed techniques [2].

Many organisations missed an opportunity to proactively manage the public mesh debate. Instead of calling physicians and industry together to discuss how the available resources could be optimally used, they remained silent. Ultimately, industry was forced to reduce its sponsorship budgets, including the support of physicians attending annual scientific meetings.

## The role of the patient

The patient is the one who has the most to lose in this debate. The patient is also the one for whom it is most difficult to determine what to believe and what not to believe. Indeed, there are many women who have suffered from an overly rapid introduction of a new surgical technique that was not yet ready to implement in daily clinical practice. In the (social) media, these women are over-represented. Although physicians say these “media” patients are not typical and not representative, our patients identify with them.

Apart from the patients with mesh complications, those who have no symptoms suffer as well. They live daily with the fear that one of those terrible complications may still happen to them. They question whether they should find a doctor who can remove their mesh. In addition, women suffering from pelvic organ prolapse, especially those with a recurrence, choose to continue to suffer from their symptoms rather than undergoing such a dangerous surgical procedure.

## The role of industry

One misconception is that industry has used doctors and patients to sell their products. If industry is not interested in selling its products, it is an unreliable partner. If we believe in the products, we should offer them to as many women as possible to their benefit, and if we have doubts about the product, we should research them better before offering them to our patients and their potential complications. Industry cannot do more than meet the requirements that physicians and professional organisation have defined as minimal standards to gain access to the market with a surgical technique. If we do not work harder to optimise those requirements, we only have ourselves to blame.

Pelvic floor implants are relatively inexpensive [3]. Worldwide, it is estimated that 90,000 perigees were sold at an average of US$ 900, which implies that US$ 81 million were earned in the lifecycle of this product. In comparison: an antimuscarinic drug generates more than US$ 20 billion over the lifetime of the patent.

It is clear that the resources available for developing and performing clinical studies, etc., for a pelvic floor implant are related to the profit that can be made. Implants for different indications such as hip implants, heart valves, neurostimulators result in significantly greater profits. The reasons for this may be speculated upon, but one reason might be that the social awareness and impact of a condition determines the willingness of society to address a medical condition and to pay for better solutions. In addition, the more profit industry can generate from a health problem, the faster the health problem is solved, and the better the available treatment options for patients.

We now live in an era where we should be happy that companies are still interested in working in the field of pelvic floor medicine. Again, we should not blame industry, but ourselves, as we have not supported those who have left the playing field or at least asked them how we can offer support. With that, not only their current commercial products, but also their budgets, research and development projects, and their innovative insights have disappeared.

The fact that industry is losing interest in pelvic floor medicine, is a threat to our future and our patients. We should seek to reverse this and make pelvic floor medicine attractive again to industry. The more interest we can generate within industry with regard to the physical problems of our patients, the faster we can bring better options to our patients.

## The role of urogynaecology organisations

Professional physician organisations should be the coordinators of all the stakeholders involved in the field of urogynaecology. This means that together with representatives of the patients (“patient organisations”) they develop a priority agenda. This agenda involves the topics that need the greatest attention, either because knowledge is insufficient, or because existing knowledge is not optimally implemented. The next step is to discuss this list with representatives of industry, patient representatives, insurance companies, and organisations with an intention to donate money to women’s health. Together, we can design a roadmap for the coming years that involves the top priority topics that need to be answered within the available budget. The professional organisations facilitate the interaction between the different stakeholders and communicate the timetable of these actions not only to their members, but also to society at large [4].

By sharing our roadmap and efforts to make progress in our field with the general public, society becomes a stakeholder as well. If unexpected adverse outcomes of vaginal mesh surgery had happened within an infrastructure as described here, society would have known that a promising surgical technique is under evaluation, and would have understood that as a result of adverse events, wide implementation of mesh surgery is not yet feasible. The reputation of urogynaecology would have benefitted from such an infrastructure. Today, we appear to be disorganised owing to a lack of coordination and leadership.

It is not too late; we need a mindshift. The relationship with industry cannot be opportunistic and unidirectional. We do not have the skills, time or energy to create new technology in combination with our work as physicians. Some of us may have good ideas, some even a brilliant ideas, but we need to collaborate from the beginning, to make sure that there is mutual benefit, otherwise there is no incentive to be involved in the field of medicine. We should not be ashamed to explain this to our patients; furthermore, we should introduce them to industry, so that industry can work on solutions that meet the demands and needs of our patients. We need to work on requirements for the safe introduction of products, and collaborate on realising these requirements. Professional organisations are the connector, the facilitator and the bridge to society, whose attention is urgently needed, to help us solve the problems of patients with pelvic floor problems worldwide, who deserve the best solutions.

## References

[1] Sliva WA, Karram MM (2005). Scientific basis for use of grafts during vaginal reconstructive procedures. Curr Opin Obstet Gynecol.

[2] Altman D, Väyrynen T, Engh ME, Axelsen S, Falconer C, Nordic Transvaginal Mesh Group (2011). Anterior colporrhaphy versus transvaginal mesh for pelvic-organ prolapse. N Engl J Med.

[3] Jacklin P, Duckett J (2013). A decision-analytic Markov model to compare the cost-utility of anterior repair augmented with synthetic mesh compared with non-mesh repair in women with surgically treated prolapse. BJOG.

[4] Luce BR, Paramore LC, Parasuraman B, Liljas B, de Lissovoy G (2008). Can managed care organizations partner with manufacturers for comparative effectiveness research?. Am J Manag Care.

